# Bloodstream infection, peritonitis, and pneumonia caused by *Pasteurella multocida* in a patient with liver cirrhosis despite no animal contact: case report and literature review

**DOI:** 10.3389/fcimb.2023.1267941

**Published:** 2023-09-26

**Authors:** Bin Lu, Xuewen Feng, Tinghua Ye, Kangfei Shan, Sipei Wang, Yunzhen Shi, Xinling Pan

**Affiliations:** ^1^ Department of Infectious Diseases, Affiliated Dongyang Hospital of Wenzhou Medical University, Dongyang, China; ^2^ State Key Laboratory for Diagnosis and Treatment of Infectious Diseases, National Clinical Research Center for Infectious Diseases, Collaborative Innovation Center for Diagnosis and Treatment of Infectious Diseases, The First Affiliated Hospital, College of Medicine, Zhejiang University, Hangzhou, China; ^3^ Department of Clinical Laboratory, Affiliated Dongyang Hospital of Wenzhou Medical University, Dongyang, China; ^4^ Department of Radiology, Affiliated Dongyang Hospital of Wenzhou Medical University, Dongyang, China; ^5^ Department of Biomedical Sciences Laboratory, Affiliated Dongyang Hospital of Wenzhou Medical University, Dongyang, China

**Keywords:** *Pasteurella multocida*, liver cirrhosis, no animal contact, bloodstream infection, peritonitis, pneumonia

## Abstract

*Pasteurella multocida* is an opportunistic pathogen. Previously reported infections associated with *P. multocida* have often been linked to contact with cats, dogs, and other animals. Cases of systemic multiple-site infections following *P. multocida* infection are rare. This case study presents a 49-year-old middle-aged man with post-hepatitis B cirrhosis and no history of animal contact. The patient was admitted with symptoms of fever accompanied by diarrhea, abdominal distension, and cough. Blood tests showed elevated levels of CRP, PCT, and IL-6, and blood culture revealed the growth of *P. multocida*. CT scans revealed a large amount of abdominal effusion, a small amount of pleural effusion, and pulmonary infection foci. The patient’s condition improved after successive administration of ceftriaxone and levofloxacin to fight the infection, and abdominal puncture and drainage. Multiple-site infections caused by *P. multocida* are rarely encountered in patients with liver cirrhosis but without animal contact, which could be regarded as serious conditions warranting careful attention in terms of clinical diagnosis and treatment.

## Introduction

*Pasteurella multocida* is an opportunistic pathogen and a bacterium that is commonly found in the respiratory and digestive tracts of many animals including poultry (Chen et al., 2002). *P. multocida* is a small, nonmotile, facultative anaerobic gram-negative coccobacillus, with a length of approximately 1.0–2.0 μm and a width of approximately 0.3–1.0 μm (Peng et al., 2019). The bacterium possesses various potential virulence factors, including a capsule, lipopolysaccharide, sialidase, hyaluronidase, and toxins (Wilkie et al., 2012). *P. multocida* can infect various hosts, including humans, livestock, and wildlife (Smith et al., 2021). Previous human cases have reported *P. multocida* causing localized cellulitis, bloodstream infections, meningitis, peritonitis, pneumonia, and endocarditis, with these infections often associated with a history of close contact with animals (Jones and Lockton, 1987; Nettles and Sexton, 1997; Raj et al., 2000; O’Neill et al., 2005; Kofteridis et al., 2009; Rondon-Berrios and Trevejo-Nunez, 2010). However, cases of multi-site infections caused by *P. multocida* are rare (Fernández-Esparrach et al., 1994; Guilbart et al., 2015; Rueda Prada et al., 2022). Herein, we present a case of multi-site infection involving bloodstream infection, peritonitis, and pneumonia caused by *P. multocida*. Remarkably, the patient had no history of animal contact, which distinguishes this case from previous case reports. Several previously reported cases of *P. multocida*-related peritonitis were associated with a history of peritoneal dialysis (Cooke et al., 2004; Rondon-Berrios and Trevejo-Nunez, 2010), whereas the patient we describe did not have underlying kidney disease or a history of peritoneal dialysis. Additionally, previous case reports have suggested that patients with underlying immunodeficiency and liver dysfunction are more susceptible to developing bloodstream infections (Weber et al., 1984; Talley et al., 2016), and these high-risk factors were also present in our patient with bloodstream infection. *P. multocida* commonly infects the respiratory tract, leading to varying degrees of respiratory infections such as pneumonia, empyema, and lung abscess (Fernández-Esparrach et al., 1994; Lion et al., 1999; Kofteridis et al., 2009), with pneumonia being the predominant manifestation in the reported cases.

## Case report

### Clinical course

A 49-year-old man presented to Affiliated Dongyang Hospital of Wenzhou Medical University on November 20, 2022 with a fever accompanied by diarrhea, abdominal distension, and cough for one day. The patient reported experiencing fever since the previous day, with a peak body temperature of 39.4°C, accompanied by four episodes of yellow watery diarrhea, significant abdominal distension, and intermittent cough with slight chest tightness. Physical examination on admission revealed mild bilateral moist rales in the lungs, abdominal distension with generalized tenderness, and positive shifting dullness. No liver tenderness or limb edema was observed. The patient had a known history of chronic hepatitis B for four years but had not received any medical treatment. All the people who lived and worked surrounding this patient were negative for infection related symptoms.

### Laboratory tests

Blood routine examination conducted on the day of admission revealed several abnormal findings. The total white blood cell count was elevated at 11.91×10^9^/L, with a high proportion of neutrophils at 92.2%. The platelet count was low at 41×10^9^/L. In addition, the patient had elevated levels of C-reactive protein at 45.91 mg/L, procalcitonin at 6.41 ng/mL, and interleukin-6 at 1254 pg/mL, indicating an inflammatory response. The prothrombin time was prolonged at 23 s, the international normalized ratio was high at 2.02, and the percentage of prothrombin activity was decreased at 38%. Liver function tests showed elevated total bilirubin at 42.1 µmol/L and decreased albumin levels at 27.2 g/L. The levels of aspartate aminotransferase and alanine aminotransferase were elevated at 97 U/L and 90 U/L, with alkaline phosphatase and gamma glutamyl transferase laying within their normal ranges. The hepatitis B DNA level was 6.1×10^6^ IU/mL. Lymphocyte subset analysis revealed a decreased total lymphocyte count at 531×10^6^/L, decreased CD3^+^ T lymphocyte count at 166.9×10^6^/L, decreased CD4^+^ T cell count at 116.9×10^6^/L, and decreased CD8^+^ T cell count at 43.0×10^6^/L.

On the day of admission, aerobic and anaerobic blood cultures (bioMérieux, Lyon, France) were performed, and gram-negative bacterial growth was observed on the third day of admission. Subsequently, species identification using VITEK matrix-assisted laser desorption/ionization time-of-flight mass spectrometry (bioMérieux) was performed, identifying *P. multocida* with an identity percentage of 99.9%. Further susceptibility testing was performed using disk diffusion method according to standards and guidelines proposed by the Clinical & Laboratory Standards Institute (https://clsi.org/standards/products/microbiology/documents/m23/). The result showed that this *P. multocida* isolate was sensitive to ceftriaxone, levofloxacin, moxifloxacin, doxycycline, ampicillin, azithromycin, penicillin, tetracycline, trimethoprim/sulfamethoxazole, chloramphenicol, amoxicillin/clavulanate, and was resistant to erythromycin (**Table 1**).

**Table 1:** Drug susceptibility of *Pasteurella multocida* by disk diffusion method.

Antibiotic agent	The diameter of inhibition zone (mm)	Phenotype
Ceftriaxone	34	S
Levofloxacin	34	S
Moxifloxacin	31	S
Doxycycline	26	S
Ampicillin	27	S
Azithromycin	30	S
Penicillin	28	S
Tetracycline	30	S
Trimethoprim/sulfamethoxazole	27	S
Chloramphenicol	30	S
Amoxicillin/clavulanate	31	S
Erythromycin	18	R

On the third day of admission, the patient underwent abdominal puncture drainage, and the collected ascites samples were sent for examination. Routine analysis of the ascitic fluid showed that the fluid was yellow and turbid. The Rivalta test yielded positive results, indicating the presence of inflammation. The nucleated cell count in the ascitic fluid was 17,157×10^6^/L. The differential cell count revealed that 88.9% of the cells were polymorphonuclear cells and 11.1% were mononuclear cells. Biochemical examination of the ascitic fluid showed an adenosine deaminase level of 3.5 U/L and a lactate dehydrogenase level of 178 U/L. Cytological examination of the ascitic fluid smear revealed a significant presence of neutrophils with toxic changes, suggestive of infectious peritonitis. The results of ascites bacterial culture, ascites tuberculosis culture, and fecal bacterial culture were all negative.

### Imaging studies

**Figure 1:**
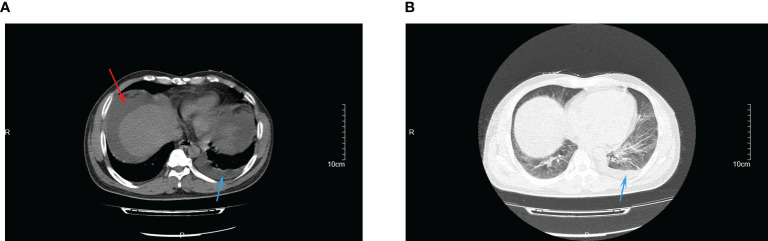
The CT images of abdominal infection **(A)** and lung infection **(B)** at admission. Red arrow indicated for ascites; blue arrow indicated for pleural effusion.

Abdominal contrast-enhanced computed tomography (CT) conducted on the second day of admission showed cirrhosis, and massive ascites in the liver (**Figure 1A**). Chest CT scan showed evidence of pulmonary infection and a small amount of pleural effusion on the left side (**Figure 1B**).

### Treatment process

**Figure 2:**
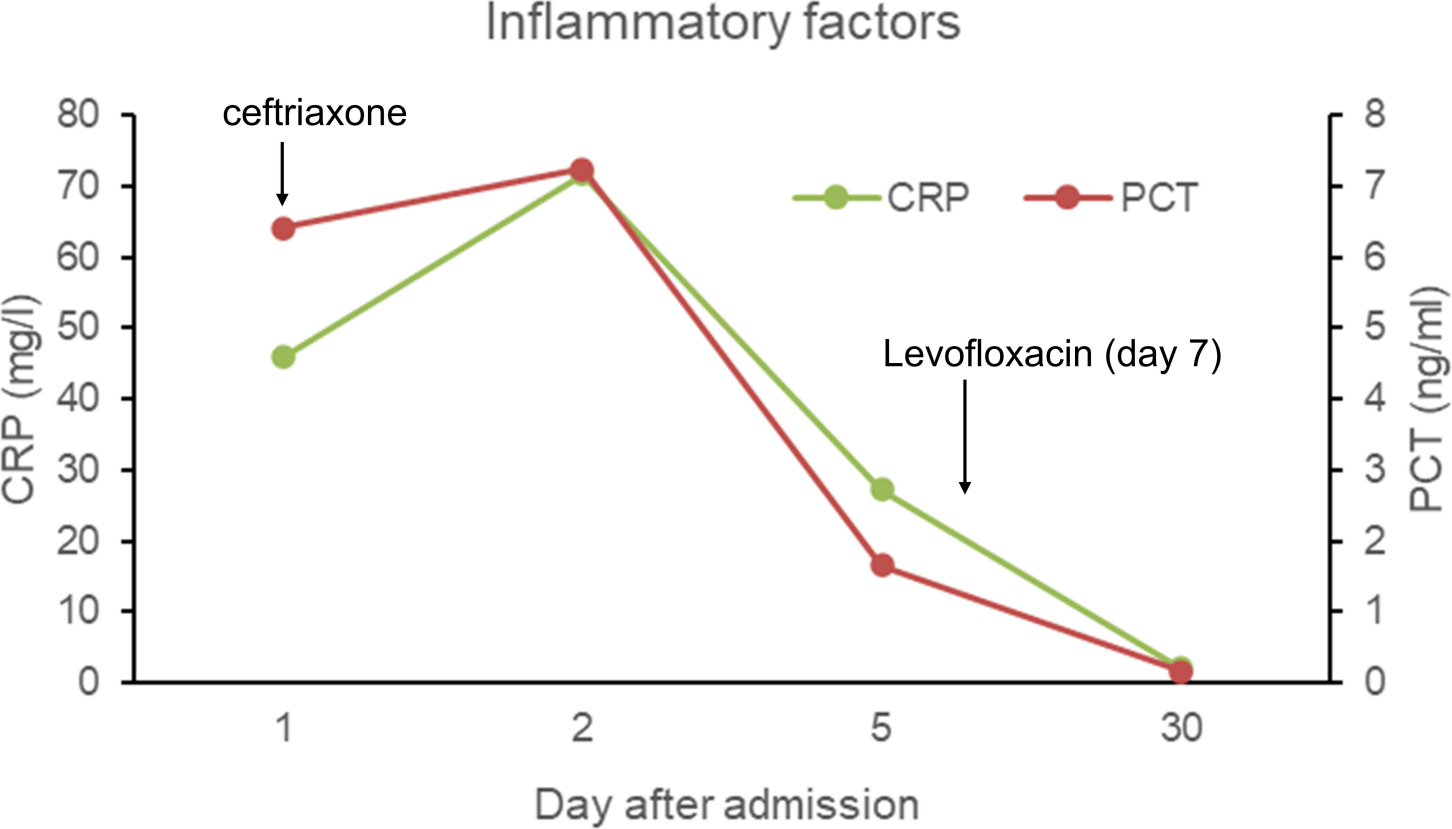
The inflammatory factors (C-creative protein and procalcitonin) in the blood throughout the treatment duration.

**Figure 3:**
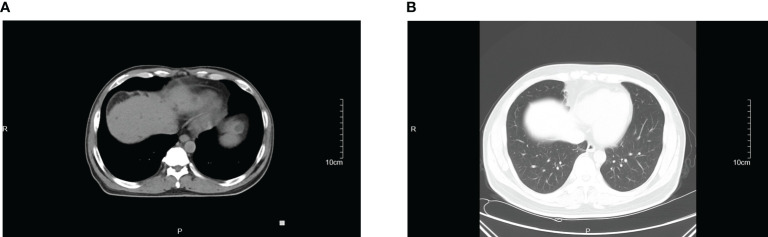
The CT images of abdomen **(A)** and lung **(B)** after treatment.

On the day of admission, the patient was started on empirical treatment with intravenous injections of 2 g of ceftriaxone once daily for antimicrobial therapy. Entecavir was administered for antiviral treatment against hepatitis B. Plasma and albumin transfusions were provided, along with hepatoprotective agents, diuretics, antidiarrheals, and cough suppressants. On the third day of admission, peritoneal puncture and ascites drainage were performed. However, the patient developed a rash during the course of treatment, raising the possibility of ceftriaxone allergy. Consequently, ceftriaxone was discontinued on the seventh day of admission, and intravenous injections of 0.5 g of levofloxacin once daily were administered for one day as an alternative antimicrobial to anti-infection therapy. On the eighth day of admission, the patient decided to transfer to the First Affiliated Hospital of Zhejiang University for further inpatient treatment. Upon admission to the new hospital, the patient continued to receive oral administration of 0.5 g of levofloxacin tablets once daily as part of the antimicrobial therapy. Abdominal effusion drainage, entecavir for antiviral treatment, plasma and albumin transfusions, hepatoprotective agents, and diuretics were also continued. After two weeks of treatment, the patient’s condition improved, and he was discharged from the hospital. He continued to take oral levofloxacin tablets for one more week. Three days after starting the antimicrobial therapy, the patient’s body temperature returned to normal, and symptoms such as diarrhea, abdominal distension, and cough improved after five days. Follow-up examinations revealed a gradual decline in inflammatory markers (**Figure 2**) and negative blood culture results. Ultimately, a CT scan indicated the disappearance of ascites (**Figure 3A**), pleural effusion, and pulmonary infection lesions (**Figure 3B**). The total duration of the anti-infection treatment was four weeks.

## Discussion

*P. multocida*, a facultative anaerobic gram-negative coccobacillus, is primarily found in the normal oropharyngeal flora of animals. Direct contact with animals or through pet ownership increases the risk of *P. multocida* infection. Most of the previously reported cases of *P. multocida* infection have been associated with bites, scratches, or close contact with cats, dogs, and other animals (Talan et al., 1999). However, it is worth noting that humans can become infected with *P. multocida* even without direct animal contact, by inhaling secretions contaminated with the bacterium, although this mode of transmission is relatively rare (Wilson and Ho, 2013). In the current case, the patient had no history of animal contact but had underlying cirrhosis. This highlights the importance for clinicians to consider the possibility of *P. multocida* infection, even in the absence of animal exposure, especially when patients have underlying immunodeficiency conditions such as cirrhosis.

Previous reports have primarily focused on *P. multocida* causing systemic single-organ infections, and only a few cases of simultaneous multi-site infections involving three or more sites in the body have been reported (Fernández-Esparrach et al., 1994; Guilbart et al., 2015; Rueda Prada et al., 2022). Patients with underlying immunodeficiency conditions such as liver cirrhosis are more prone to acute liver dysfunction, which is more likely to cause bloodstream infections (Weber et al., 1984; Talley et al., 2016). Our reported case involves a patient with underlying cirrhosis and severe liver dysfunction, which shares similar high-risk factors for developing bloodstream infections to those reported in some previous cases. Most cases of peritonitis caused by *P. multocida* infection have been reported in the context of peritoneal dialysis (Cooke et al., 2004; Rondon-Berrios and Trevejo-Nunez, 2010), although a few cases of peritonitis without peritoneal dialysis have been reported (Vakil et al., 1985; Fernández-Esparrach et al., 1994). In our case, the patient had underlying cirrhosis but no kidney disease or history of peritoneal dialysis, which is uncommon. *P. multocida* colonizes the respiratory tract, and pneumonia, empyema, and lung abscess have been reported as manifestations in previous cases (Fernández-Esparrach et al., 1994; Lion et al., 1999; Kofteridis et al., 2009). In our case, the main manifestation was pneumonia, accompanied by a small amount of pleural effusion, but it did not progress to severe empyema or lung abscess. A similar case was previously reported involving a patient from a hospital in Barcelona, Spain, with underlying liver cirrhosis who was admitted due to *P. multocida* infection resulting in bloodstream infection, peritonitis, and empyema. This patient had no history of peritoneal dialysis but had a previous cat scratch and eventually developed progressive liver failure and died. In our case, the patient developed bloodstream infection, peritonitis, and pneumonia after *P. multocida* infection, despite no history of animal exposure. However, the patient had a favorable outcome after active treatment, highlighting the fact that *P. multocida* can cause multi-organ infections in immunocompromised patients. Without timely and aggressive treatment, these infections can lead to systemic organ dysfunction and even death.

The patient in this case had underlying cirrhosis, and the results of lymphocyte subpopulation analysis showed the total number of lymphocytes and the absolute number of CD4^+^ T cells and CD8^+^ T cells decreased significantly, indicating immunodeficiency. Previous studies have shown that *P. multocida* infections are often associated with T-cell-mediated immune damage (Hildebrand et al., 2015), and the decrease in T-lymphocyte subpopulation count and function may have contributed to the multi-site infection in the patient. Cytological examination of the ascitic fluid in this patient indicated peritonitis caused by *P. multocida*, although ascites cultures yielded negative results. This could be attributed to the early use of effective antibiotic therapy and the delayed ascites culture (performed on the third day of admission). In addition, conventional bacterial cultures may have lower sensitivity compared to next-generation sequencing in diagnosing bacterial infections in abdominal fluid (Goelz et al., 2021), especially when patients have received early antibiotic treatment, highlighting the limitations of standard bacterial cultures (Yoo et al., 2020).

Furthermore, the patient presented with a cough and slight chest tightness, and moist rales were detected upon auscultation of the lungs at admission. Pulmonary infection foci and pleural effusion were observed; however, owing to the minimal amount of pleural effusion, no thoracentesis or drainage was performed. Fortunately, with effective antimicrobial treatment, the pulmonary infiltrates and pleural effusion resolved, suggesting that the primary concern was pneumonia and pleural effusion caused by *P. multocida* infection. The drug sensitivity testing of *P. multocida* in this case revealed sensitivity to penicillin, cephalosporins, quinolones, and other commonly used antibiotics, which is consistent with previous case reports (Rondon-Berrios and Trevejo-Nunez, 2010; Abdelkarim et al., 2014; Katechakis et al., 2019; Hanson et al., 2022). Upon admission, ceftriaxone was chosen as the empirical antimicrobial treatment, and subsequent drug sensitivity testing confirmed its effectiveness. However, owing to the development of a rash in the patient, ceftriaxone allergy was considered, leading to a switch to levofloxacin for anti-infective therapy. In addition, performing ascites drainage while undergoing antibiotic therapy was a crucial measure for the success of treatment in this case.

## Conclusion

In conclusion, we presented a case of a patient with liver cirrhosis who developed a multi-site infection with *P. multocida*, resulting in bloodstream infection, peritonitis, and pneumonia, despite no history of animal exposure. The patient received antimicrobial therapy with ceftriaxone followed by levofloxacin and underwent concurrent paracentesis drainage. Subsequently, his body temperature returned to normal, and both the ascites and pulmonary infiltrates resolved, resulting in a favorable prognosis. This case demonstrates that the use of effective antibiotics, along with local drainage intervention, is crucial for the successful treatment of patients with multi-site invasive infections caused by *P. multocida*.

## Data availability statement

The original contributions presented in the study are included in the article/supplementary material. Further inquiries can be directed to the corresponding author.

## Ethics statement

The studies involving humans were approved by the Ethics Committee of Affiliated Dongyang Hospital of Wenzhou Medical University. The studies were conducted in accordance with the local legislation and institutional requirements. The participants provided their written informed consent to participate in this study. Written informed consent was obtained from the individual(s) for the publication of any potentially identifiable images or data included in this article. Written informed consent was obtained from the participant/patient(s) for the publication of this case report.

## Author contributions

BL: Data curation, Methodology, Writing – original draft, Writing – review & editing. XF: Investigation, Resources, Writing – original draft. TY: Methodology, Validation, Writing – original draft. KS: Resources, Visualization, Writing – original draft. SW: Formal Analysis, Resources, Writing – original draft. YS: Writing – original draft, Conceptualization, Funding acquisition. XP: Writing – original draft, Methodology, Software, Visualization, Writing – review & editing.
